# Needs assessment for master of nursing program among Kenyan nurses

**DOI:** 10.1371/journal.pone.0322813

**Published:** 2025-05-05

**Authors:** Stevenson K. Chea, Dredah Mwadulo, Abednego Kioko, Lucy Kiriba, Martin Mulala, Isaac Kyalo, Everlyne Shitoyi, Elizabeth Mutunga, Mwaswere Juma, Nickcy Mbuthia

**Affiliations:** 1 Department of Nursing Sciences, School of Health and Human Sciences, Pwani university, Kilifi, Kenya; 2 Department of Public Health and Primary Care, Faculty of Medicine and Health Sciences, Ghent University, Ghent, Belgium; 3 Department of Health Sciences, School of Pure, applied and Health Sciences, Maasai Mara university, Narok, Kenya; 4 Department of Nursing, School of Nursing and Public Health, Chuka University, Chuka, Kenya; 5 Department of Medical-Surgical Nursing and pre-clinical services, School of Health Sciences, Kenyatta University, Nairobi, Kenya; University of Vermont, UNITED STATES OF AMERICA

## Abstract

**Background:**

Nurses comprise the dominant cadre of healthcare workers yet there remains an acute shortage of nurses globally with Africa most affected. However, access to higher nursing education in sub-Saharan Africa remains limited. We aimed to i) Assess the need for a Master of Nursing (MScN) program among graduate nurses in Kenya ii) Identify preferred MScN program options among graduate nurses intending to enrol for MScN in Kenya iii) Identify skills mismatch among graduate nurses in Kenya iv) Assess the relationship between intention to enrol in MScN program and job satisfaction among graduate nurses in Kenya.

**Methods:**

A cross-sectional design employing an online survey was used. Consenting nurses with a first degree in nursing were included. Socio-demographic indicators, job satisfaction and skills mismatch were assessed. The need for an MScN program was assessed by determining the proportion of participants who expressed the desire to pursue an MScN program. Preferred MScN program options were determined as frequencies and proportions. Skills mismatch was computed as frequencies and proportions. The relationship between the need for MScN and job satisfaction was assessed using the point biserial-correlation.

**Results:**

Of the 355 volunteers enrolled, (n = 337, 94.9% [95% CI: 92.1–96.9]) expressed the desire to pursue MScN training with the critical care/renal specialty (n = 84, 24.9% [95% CI: 20.3–29.9]) being the most preferred. A majority of the volunteers (n = 319, 89.9%) felt their skills were inferior to their responsibilities (under skilled). We found no significant correlation between the need for MScN and job satisfaction (r = 0.058; p = 0.269).

**Conclusion:**

Our findings suggest a strong desire by graduate nurses in Kenya to pursue MScN with a preference for critical care specialization. There is a need to establish more MScN programs in Kenya coupled with the deployment of nurses as per the scope of practice.

## Background

Human resources for health is one of the six building blocks of any healthcare system [1]. As the current push in sub-Saharan Africa to achieve universal health coverage continues, in line with sustainable development goal (SDG) 3, there is a need to train human resources with the required skills and competencies [2]. One of the key human resources in health is the nursing workforce [3,4]. Nurses comprise the dominant cadre of healthcare workers yet there remains an acute shortage of nurses globally [3,5]. Specifically, the World Health Organization (WHO) estimates that about 27 million individuals make up the global nursing workforce accounting for approximately 50% of the global health workforce [3]. Further, the WHO estimates that nurses account for over 50% of the global shortage of healthcare workers with the largest needs-based shortage of nurses being experienced in sub-Saharan Africa [3]. Despite the general increase in number of nurses in Kenya over the years, there were 159.3 nurses per 100,000 population in 2013 against a World health Organization recommendation of 228 per 100,000 population [6]. By 2020, there were an estimated 109,659 nurses in Kenya with varying qualifications ranging from certificate to degree [7]. At the same time, the density of health professionals including nurses, per 10,000 population was 30.1 which represents about 68% of the sustainable development goals threshold index of 44.5 health professionals per 10,000 population [7]. From 2006–2015, 3,849 students were admitted into the bachelor of science in nursing (BScN) degree program in Kenya representing 12.4% of all students enrolled into various nursing programs in that period [6]. The 3,849 translates to approximately 385 BScN students enrolled into training annually [6]. By 2020 there were 7,242 nurses with a BScN which represented a 149% increase from 2,907 in 2015 [8]. Data on estimates of nurses who are MScN or PhD holders remain scanty as the Nursing Council of Kenya (NCK),the government body mandated to regulate nursing practice in Kenya, does not register or licence nurses holding these higher qualifications [8]. Despite the increasing demand for highly qualified nursing professionals from the employers, and the growth in nursing education, there are challenges that continue to impact on the training of these professionals including outdated curricula, shortage of faculty, under-qualified faculty, underfunding of institutions and limited teaching and learning resources [8]. Despite the increase in bachelor’s degree-qualified nurses, they account for only 7.7% of the nurses in Kenya [9]. The Kenyan Ministry of Health Human Resources for Health Norms and Standards Guideline [10] stipulates the workload for nurses with diploma qualification but remains silent on graduate nurses. This may partially explain the low absorption of graduate nurses into the labor market.

Highly trained nurses are a critical human resource for health [3]. Evidence suggests that nurses with advanced academic qualifications perform better compared to their counterparts on several indicators of quality of care including patient health outcomes [11–13]. However, access to higher nursing education in sub-Saharan Africa remains limited which may partially explain the shortage of this specific cadre of nurses in this setting [3]. In Kenya, there are 74 universities (31 of which are government-funded) and only a few of these offer Master of Science in Nursing (MScN) programs [14]. Example, within the six Coastal counties of Kenya, there are two public universities [14] none of which offer MScN training.

Apart from better professional performance, advanced nursing academic qualifications have been associated with job satisfaction [15]. Specifically, the desire to enrol in advanced nursing studies has been predicted by low job satisfaction [15]. One of the links between job satisfaction and higher education achievements is job performance [16]. Higher academic qualifications lead to improved job performance [13]. On the other hand, employees who shine in their professional roles feel more valued hence increased job satisfaction [16]. Importantly, nurses’ job dissatisfaction either due to low academic qualifications or other factors has been shown to compromise the quality of care and consequently patient safety [17].

One of the ways to increase access to postgraduate nursing education is to have more institutions offering such programs. However, to start a new MScN program, it is important first to assess its demand among the nurses themselves. Previous training needs assessments in Kenya highlighted the need for highly trained nurses who can engage in advanced nursing practice [18,19]. However, these training needs assessments did not specifically address the demand for the MScN program and the preferred program options. We aimed to i) Assess the need for an MScN program among graduate nurses in Kenya ii) Identify preferred MScN program options among graduate nurses intending to enrol for MScN in Kenya iii) Identify skills mismatch among graduate nurses in Kenya iv) Assess the relationship between intention to enrol in MScN program (educational need for MScN) and job satisfaction among graduate nurses in Kenya.

## Methods

### Design and population

A cross-sectional design employing an online survey was used. Consenting nurses or nurse interns with a first degree in general nursing or any of the nursing speciality areas including BSc in Midwifery were included. Nurses whose highest qualification was above or below a bachelor’s degree were not eligible to participate. Nurses who were enrolled in a master of science in Nursing (MScN) program at the time of data collection were equally excluded. Including nurses already enrolled in MScN would likely introduce bias as they are already enrolled so would likely not express a desire to pursue MScN. Sample size estimation was done using the G-power software version 3.1.9.7 (Universität Kiel, Germany). With a small effect size of 0.14 [20–22], power 80% and significance level of 0.05% a sample size of 350 was deemed adequate.

### Sources of data and measures

#### Socio-demographic characteristics.

Socio-demographic data were collected using a questionnaire prepared for this study and included age, sex, religion and marital status. Others included the need for MScN and MScN program preferences (S1 File). The need for MScN was defined as expression of a desire to pursue MScN.

#### Job satisfaction.

The Ward Organizational Features Scale (WOFS) [23] was used to assess job satisfaction. The scale is reliable in assessing job satisfaction (Cronbach’s alpha 0.77)[23]. The scale comprises seven items each answered on a 5 – point likert scale ranging from strongly disagree (scored 1 point) to strongly agree (scored 5 points). A higher total score indicates a higher level of job satisfaction (S1 File).

#### Skills mismatch.

Two questions from the Program for the International Assessment of Adult Competencies (PIAAC) background questionnaire [24] were used to assess skills mismatch. The two questions were i) Do you feel that you have the skills to cope with more demanding duties than those you are required to perform in your current job? (Yes [] No []) ii) Do you feel that you need further training in order to cope well with your present duties? (Yes [] No []). Consequently, three meaningful aspects of skills mismatch were determined: a) Over-skilled defined as the possession of skills that are superior compared to those needed for a participant’s job [24]. Education qualification was used as a proxy for skills such that higher qualifications meant higher skills [25]. A volunteer was over-skilled if he/she responded “yes” to question 1 and “no” to question 2 and/or “yes” to both questions; b) Well-matched defined as possession of skills that are just adequate to enable the volunteer to perform in his/her job. A volunteer was well-matched if he/she responded “no” to both questions; c) Under-skilled defined as the possession of skills that are inferior to the volunteer’s current job. A volunteer was under-skilled if he/she responded “no” and “yes” to question 1 and 2 respectively and/or “yes” to both questions. A fourth aspect known as “over-skilled as well as under-skilled” was also generated due to overlap in responses to the questions but this was not used in analysis since it is not meaningful [24]. To take care of this omission, the final number of over-skilled volunteers was determined by subtracting the number of under-skilled volunteers from the over-skilled category (S1 File).

### Development of the online survey

The survey was prepared on google forms and organized into sections 1–25. Section one carried the title of the study. Sections two to nine carried the introduction and eligibility checks. The introduction part provided the names of the main investigator. Further, the aims of the study were explained including details about the ethical clearance that had been obtained to allow the study to proceed. For the eligibility checks, each question was in a section of its own. For each question, if the participant’s response did not fulfil the criteria for eligibility, they were prompted to submit the form as they were not eligible. Participants whose responses to the eligibility questions were in the affirmative proceeded to sections ten and eleven for informed consenting. This section explained that the participant was eligible and that he/she was now requested to indicate if he/she was willing to take the survey. By selecting the “Yes” option, the participant was deemed to have given consent to participate in the survey. Sections twelve to twenty-five contained the survey questions. To minimize non-response and missing data, all questions including eligibility checks were mandatory thus a participant would be prompted to fill in missing data upon submitting the survey. To minimize instances of participants taking the survey twice, one could not take the survey more than once from the same account. Cookies were not used to assign a unique user identifier to each client computer neither was the internet protocol address of the participant computer used to identify potential multiple entries. Adaptive questioning was used where some survey questions were conditionally displayed depending on responses to other items. This approach helped reduce the number of questions as well as the complexity of the survey. To enhance the survey completion rate, an average of two to three questions were included on each page of the survey form totaling to 39 questions. To test the usability and technical functionality of the electronic questionnaire, the investigators did a dry run of the survey and made necessary adjustments to ensure everything was okay. Respondents were able to review and change their answers by pressing on the back button.

### Procedure

During data collection, data collection tools (complete with eligibility criteria and informed consent forms) were prepared on Google Forms and circulated on email and social media platforms (WhatsApp and Facebook) to nurses and nurse interns all over Kenya. Specifically, a link to the survey was shared on the face book page of the Kenya National Union of Nurses (KNUN). KNUN is a trade union that advocates for the welfare of member nurses in Kenya. All nurses regardless of qualification are eligible to join. The face book page is visited by nurses with various qualifications including those who are not members of KNUN. Nurses visit the page seeking to update themselves on events affecting nursing in Kenya among other reasons. Though posting the link to the survey on this face book page ensured wider reach of the nursing fraternity as any nurse accessing the internet could participate in the survey, nurses who do not frequently use social media may have been disadvantaged posing a risk of bias. Email contacts of nurses and nurse interns were obtained through nurse managers in some of the hospitals and a link to the questionnaire was shared. Nurse managers were then requested to circulate the link to nurses and nurse interns in their facilities as well as to other nurses and nurse interns they may be in contact with to ensure a wider reach to the nurses. A minimum of 20 nurse managers spread across 13 health facilities were requested to circulate the link to the survey. Being an open online survey, we did not assess representativeness of the sample after close of data collection. However, we took steps to minimize the risk of having an unrepresentative sample: i) the nurse managers requested to circulate the link to the survey were distributed across 13 facilities in 8 counties ii) the investigators, who are faculty in their respective institutions, shared the link with their alumni who were interns that time in various health facilities all over Kenya. The interns also shared with other nurses within their facilities.

Any eligible nurse/nurse intern with the link was able to participate, thus it was an open survey. In addition, nurses/nurse interns whose workstations were within reach of the investigators were contacted directly and requested to provide their email addresses so that the link could be shared. They were equally requested to share the link with their contacts. Since the survey was prepared on google forms, participants responses were captured automatically as they completed the survey. Though the link to the survey was shared on whatsapp groups and face book, it was not mandatory for nurses visiting the face book page or members of the whatsapp groups to participate. No incentives were offered for participating in the survey. Data collection started on 8^th^ May 2023 and ended on 6^th^ December 2023.

### Data analysis

First, a description of volunteers’ socio-demographic characteristics using frequencies and proportions was done and presented in a table. Secondly, the need for an MScN program was assessed by determining the proportion of volunteers who expressed a desire to pursue the MScN program, corresponding 95% confidence intervals were presented. Thirdly, to assess preferred MScN program options, frequencies and proportions of volunteers opting for the various program options including area of specialization and mode of study were presented with their corresponding 95% confidence intervals. Next, skills mismatch was computed as frequencies and proportions with their corresponding 95% confidence intervals. Finally, the association between the need for an MScN and job satisfaction was assessed. First, job satisfaction was determined where for each volunteer, the score in each of the 7 items in the WOFS was added to get the overall score of job satisfaction for the volunteer. Scores of all volunteers were then added to get the total score. The mean score was the total score divided by number of volunteers, the mean score and its standard deviation were presented. Then, to assess the relationship between the need for MScN and job satisfaction, the point biserial correlation was used because the need for MScN is a dichotomous variable. P value and the correlation coefficient were presented. Analysis was performed in Stata 15.0 (StataCorp.2017. Stata Statistical Software: Release 15. College Station, TX: StataCorp LLC. 2019) and graphs were generated using GraphPad Prism version 8.0.2 (GraphPad Software, California).

### Ethics

The study received ethical clearance from the Pwani University Institutional Scientific and Ethics Review Committee (ISERC/PU-STAFF/001/2023). Written informed consent was obtained from all participants. Specifically, the first page of the survey indicated the name of the main investigator and his affiliation. In addition, the aim of the survey was outlined followed by eligibility checks. After completing the eligibility checks, eligible participants proceeded to the informed consent page. On this page it was explained that participation in the survey was voluntary and it was going to involve completing the survey. Further, it indicated that completing the survey would take approximately half an hour and that participant’s responses would not be shared with anyone outside the study team. Finally, an email address of one of the investigators was provided for participants to use should they have questions or concerns. Since personal information like age and sex was collected, unauthorized access to such information was minimized as the survey was prepared on the email account of the lead investigator thus only him had the log in details.

## Results

### Characteristics of graduate nurses enrolled into the study [n = 355]

**Fig 1:**
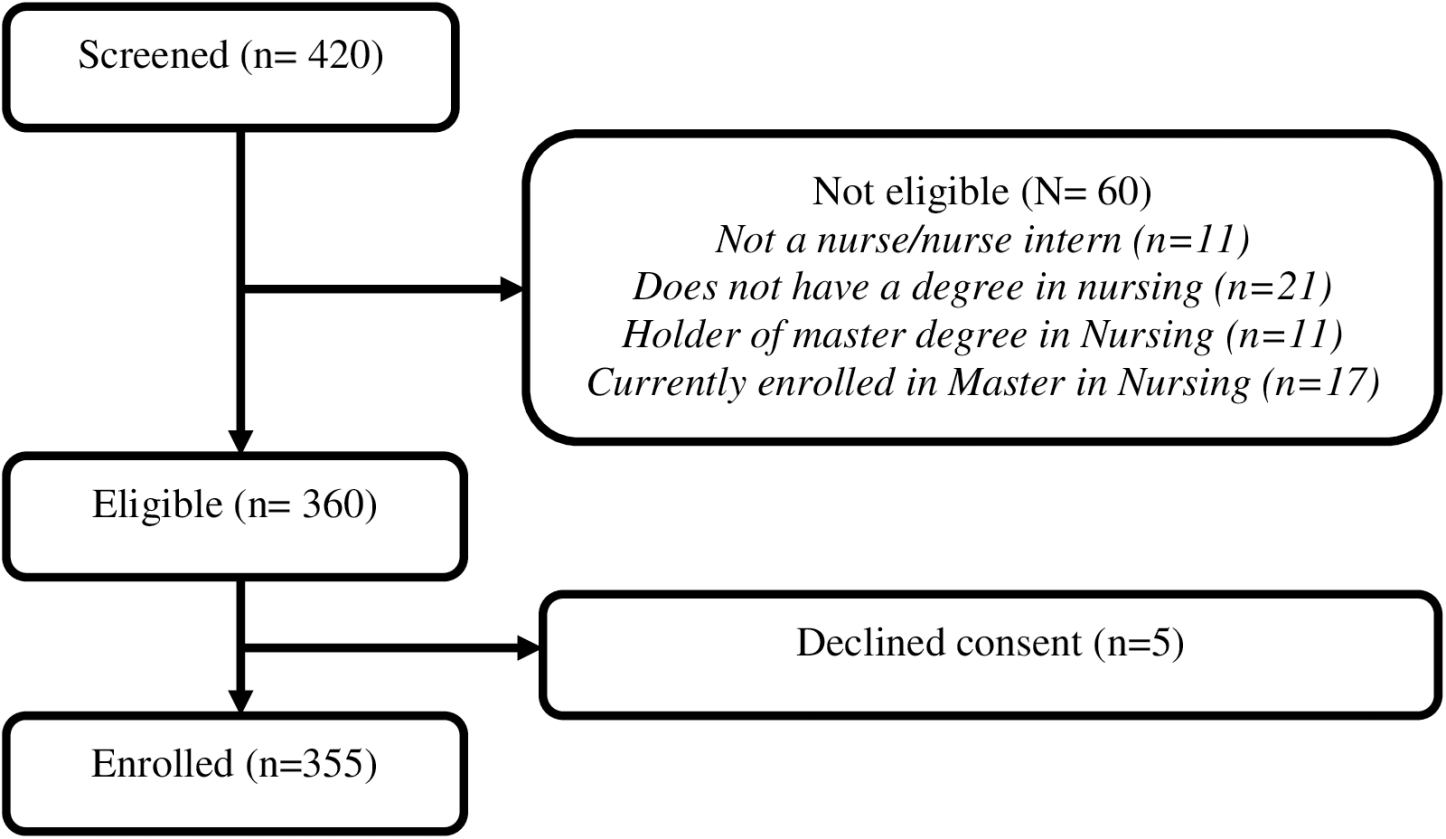
Flow diagram showing enrolment of graduate nurses in Kenya.

A total of 420 nurses were screened out of which 360 met the eligibility criteria. Of these, 5 declined consent hence 355 were enrolled into the study (Fig 1). Further exploration of our data shows that the enrolled participants were drawn from 44 out of the 47 counties in Kenya suggesting that the sample was representative. Further, all individuals who agreed to participate in the survey submitted the last page of the survey resulting to a completion rate of 100%. Since all survey questions were mandatory, there was no minimal missing data thus all questionnaires were included in analysis. No questionnaires were excluded from analysis based on the duration of time taken by the participant to complete the survey. The mean age [standard deviation] of the volunteers was 26.3 [9.3] years. Majority of the volunteers were aged above 24 years (n = 235,66.2%), Christians (n = 306, 86.2%), single/divorced/seperated (n = 220, 62.0%) and non interns (n = 187, 52.7%). A further majority (n = 338, 95.2%) felt there was need to start more MScN programs in Kenya and that there was a skills gap that MScN training would fill (n = 333, 93.8%). Among volunteers that were non interns and employed, majority were working in public facilities (n = 94, 56.9%), did not have administrative duties at their place of work (n = 100, 60.6%), had a working experience of less than five years (n = 64, 38.8%) and were working in the Medical-surgical department (n = 107, 64.9%). Among volunteers who were working, majority had at least one colleague with MScN training (n = 188, 55.1%), believed the organization they worked for would benefit if more MScN graduates were employed (n = 318, 93.3%), felt they needed further training to cope with their current duties (n = 306, 89.7%) and believed nurses with inferior qualifications were carrying out duties that ought to be a preserve of those with MScN training (n = 165, 48.4%) [Table 1].

**Table 1:** Characteristics of graduate nurses in Kenya (n = 355).

Characteristic	Category	Male (n = 175)	Female (n = 180)	Overall (n = 355)
Age (years) M/SD	–	26.4 (7.6)	26.2 (10.7)	26.3 (9.3)
Age group (Years)	≤24	55 (31.4)	65 (36.1)	120 (33.8)
	>24	120 (68.6)	115 (63.9)	235 (66.2)
Religion	Christian	153 (87.4)	153 (85.0)	306 (86.2)
	Muslim	11 (6.3)	24 (13.3)	35 (9.9)
	Others	11 (6.3)	3 (1.7)	14 (3.9)
Marital status	Married	59 (33.7)	76 (42.2)	135 (38.0)
	Single/divorced/separated	116 (66.3)	104 (57.8)	220 (62.0)
Training trajectory	Bachelor’s degree direct	165 (94.3)	145 (80.6)	310 (87.3)
	Diploma to Bachelor’s degree	10 (5.7)	35 (19.4)	45 (12.7)
Intern nurse?	Yes	90 (51.4)	78 (43.3)	168 (47.3)
	No	85 (48.6)	102 (56.7)	187 (52.7)
Employment status	Unemployed	9 (5.1)	5 (2.8)	14 (3.9)
	Part-time/intern	103 (58.9)	91 (50.6)	194 (54.7)
	Full-time/Self-employed	63 (36.0)	84 (46.6)	147 (41.4)
*Employer	Faith-based	6 (8.5)	7 (7.5)	13 (7.9)
	Private	28 (39.4)	30 (31.9)	56 (35.2)
	Public	37 (52.1)	57 (60.6)	94 (56.9)
*Administrative duties	Yes	28 (39.4)	37 (39.4)	65 (39.4)
	No	43 (60.6)	57 (60.6)	100 (60.6)
*Work experience (Years)	<5	36 (50.7)	28 (29.8)	64 (38.8)
	5 - 10	25 (35.2)	33 (35.1)	58 (35.1)
	>10	10 (14.1)	33 (35.1)	43 (26.1)
*Employment terms	Contract	38 (53.5)	43 (45.7)	81 (49.1)
	Permanent	33 (46.5)	51 (54.3)	84 (50.9)
*Working department	Medical-surgical	43 (60.6)	64 (68.1)	107 (64.9)
	Obstetrics/midwifery	17 (23.9)	16 (17.0)	33 (20.0)
	Administration/community	11 (15.5)	14 (14.9)	25 (15.1)
Skills mismatch	Under skilled	159 (90.9)	160 (88.9)	319 (89.9)
	Over skilled	15 (8.6)	19 (10.6)	34 (9.6)
	Well matched	1 (0.6)	1 (0.6)	2 (0.6)
Job satisfaction (M/SD)	–	24.9 (4.6)	25.0 (4.6)	25.0 (4.5)

*N = 165; Excludes interns (n = 168), unemployed nurses (n = 14) and self-employed nurses (n = 8)

M (Mean); SD (Standard deviation).

Skills mismatch (Assessed using two questions from the Program for the International Assessment of Adult Competencies [PIAAC] background questionnaire; Over-skilled is defined as possession of superior skills compared to those needed for a volunteer’s job; Well-matched is defined as possession of skills that match those needed for a volunteer’s job; Under-skilled is defined as possession of skills that are inferior to the volunteer’s current job.

Job satisfaction (Assessed using the ward organizational features scale [WOFS]; Higher score means higher job satisfaction [scores range from 7–35]).

Medical surgical (includes general nursing, palliative care, theatre, newborn unit and paediatrics).

Faith based (Religious organizations that establish and run hospitals).

Intern nurse (Nurses who have completed their bachelor’s degree training in nursing and are working under the supervision of senior colleagues for one year as a requirement for full registration as nurses in Kenya).

#### Need for MScN.

**Fig 2:**
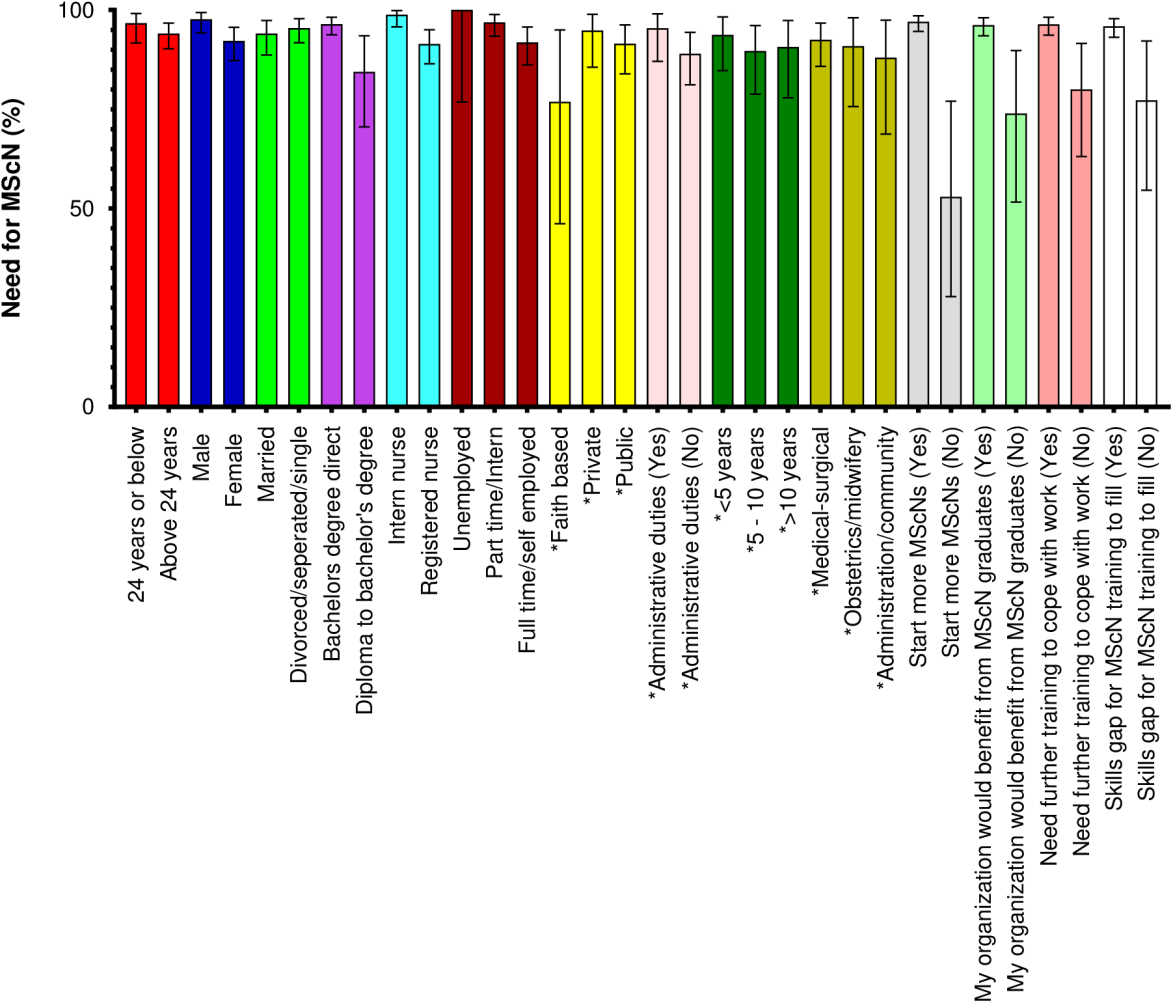
Distribution of need for MScN by selected characteristics among graduate nurses in Kenya (n = 355). MScN (Master of Science in Nursing); Bachelor’s degree direct (Nurses who directly enrolled for a degree in nursing after high school); Diploma to bachelor’s degree (Nurses who first studied nursing at a diploma level then upgraded to bachelor’s level); Intern nurse (Nurses who have completed their bachelor’s degree training in nursing and are working under the supervision of senior colleagues for one year as a requirement for full registration as nurses in Kenya). *N = 165; Excludes intern nurses (n = 168), unemployed nurses (n = 14) and self-employed nurses (n = 8).

Overall, (n = 337, 94.9% [95% CI: 92.1–96.9]) of volunteers expressed the desire to pursue MScN training with a majority, (n = 212, 62.9%), intending to do so within two years. Compared to female volunteers, a higher proportion of male volunteers expressed a desire to pursue MScN (92.2% vs 97.7%; x^2^ = 5.5604; p = 0.018). Compared to volunteers who first studied nursing at diploma level then upgraded to degree, a bigger proportion of volunteers who directly enrolled for a degree in nursing after high school expressed a desire to pursue an MScN (84.4% vs 96.4%; x^2^ = 11.7701; p = 0.001). Compared to volunteers who were practicing post internship, a higher proportion of volunteers who were still on internship expressed a desire to pursue MScN (91.4% vs 98.8%; x^2^ = 9.9747; p = 0.002). Compared to volunteers who felt there was no need to start more MScN programs in Kenya, a higher proportion of those who felt there was need to start more MScN programs expressed a desire to pursue MScN (52.9% vs 97.0%; x^2^ = 65.3994; p < 0.001). Equally, compared to volunteers who felt the organization they were working for would not benefit from employing MScN graduates, a higher proportion of volunteers who felt the organization they worked for would benefit from employing MScN graduates expressed a desire to pursue MScN (73.9% vs 96.2%; x^2^ = 21.3582; p <0.001). Likewise, compared to volunteers who felt they did not need further training to cope with their current duties, a higher proportion of volunteers who felt the need for further training to cope expressed a desire to pursue MScN (80.0% vs 96.4%; x^2 ^= 16.9057; p < 0.001). Lastly, compared to volunteers who felt there was no skills gap for an MScN training to fill, a higher proportion of those who felt that a skills gap existed expressed a desire to pursue MScN (77.2% vs 95.9%; x^2^ = 15.1910; p < 0.001) (Fig 2).

#### Preferred MScN program options.

**Fig 3:**
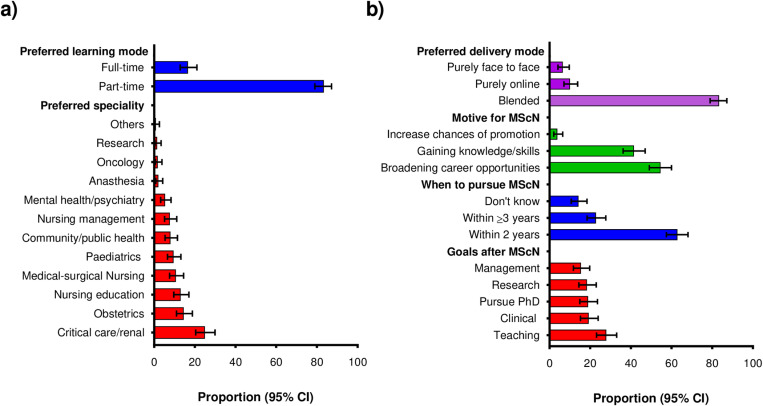
Preferences for MScN program among volunteers who expressed the desire to pursue MScN [n = 337] a) Preferred MScN speciality and preferred learning mode b) Preferred delivery mode; motive for pursuing MScN; when to pursue MScN; goals after MScN [n = 337].

Among volunteers who expressed the desire to pursue MScN, majority preferred to study the critical care/renal speciality and preferred the part-time mode of learning: (n = 84, 24.9% [95% CI: 20.3–29.9]) and (n = 281, 83.3% [95% CI: 78.9–87.1]) respectively (Fig 3a). The second most preferred speciality was obstetrics (n = 49, 14.5% [95% CI: 10.9–18.7]). Furthermore, a majority intended to pursue an MScN within 2 years from the time of enrolment into the study and preferred the blended mode of content delivery that combines face-to-face and online learning sessions: (n = 212, 62.9% [95% CI: 57.5–68.0]) and (n = 281, 83.3% [95% CI: 78.9–87.1]) respectively (Fig 3b).

#### Skills mismatch among graduate nurses in Kenya (n = 355).

**Fig 4:**
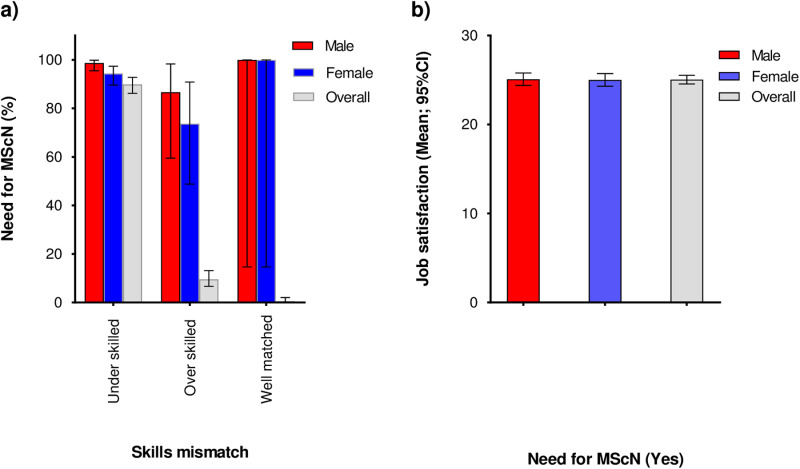
a) Skills mismatch by need for MScN among graduate nurses in Kenya (n = 355); b) need for MScN by job satisfaction (n = 337). Skills mismatch measured using two questions from the Program for the International Assessment of Adult Competencies (PIAAC) background questionnaire. Over-skilled (Possession of skills that are superior compared to those needed for a participant’s job). Well-matched (Possession of skills that are just adequate to enable the participant perform in his/her job). Under-skilled (Possession of skills that are inferior to the participant’s current job). Job satisfaction measured using the ward organizational features scale (WOFS). Higher scores represent higher job satisfaction.

Overall, a majority of the volunteers (n = 319, 89.9%) felt their skills were inferior to their responsibilities (under-skilled) while (n = 34, 9.6%) felt they were over-skilled (Table 1). Compared to volunteers who felt they were over-skilled, a bigger proportion of those who felt under-skilled expressed a desire to pursue an MScN (Fig 4a). There were no significant differences by sex in the desire to pursue MScN by skills mismatch (Fig 4a). Further, (n = 333, 93.8%) of the volunteers felt there was a nursing skills gap that would be resolved by MScN graduates. All volunteers had carried out at least one task that ought to be carried out by MScN graduates as per the nurses’ scope of practice in Kenya with “taking up the role of a consultant nurse” being the most common task (n = 18, 5.1%).

### Job satisfaction and the relationship between need for MScN and job satisfaction among graduate nurses in Kenya (n = 355)

The mean (standard deviation) of job satisfaction among the volunteers was 25.0 (4.5) (Table 1). The WOFS was found to be reliable (Cronbach’s alpha = 0.7305). Overall, job satisfaction did not differ by need for MScN (Job satisfaction, mean [standard deviation]: 23.8 [4.6] *vs* 25.1[4.5]; p = 0.865) for those who did not express the desire to pursue MScN and those who did respectively. Equally, job satisfaction among volunteers who expressed the desire to pursue MScN did not differ by sex (Fig 4b). We found no significant correlation between the need for MScN and job satisfaction (r = 0.058; p = 0.269).

## Discussion

Our findings show that about 9 out of every 10 volunteers intended to pursue an MScN. The preferred MScN speciality was critical care/renal nursing while part-time was the preferred learning mode. About 9 in every 10 volunteers felt their skills were inferior to their professional responsibilities. We found no correlation between intention to pursue MScN and job satisfaction.

About nine out of every 10 volunteers expressed a desire to pursue an MScN suggesting the need for an MScN program. A Kenyan qualitative study that explored views of stakeholders on advanced studies in nursing equally reported that a master’s program in community health nursing was needed to improve the knowledge and skills of nurses [19]. Another study conducted in Swaziland equally showed that there was a need for MScN [26]. The almost universal desire to pursue MScN in our study is suggestive of a high demand for MScN which further suggests a potential need to establish additional MScN programs in Kenya. Indeed, further exploration of our data already shows that about 95.2% of the volunteers felt there was a need to establish more MScN programs in Kenya. The need to establish more MScN programs in Kenya is further illustrated by the fact that out of the 74 chartered universities in Kenya, only 11 offer MScN out of which 6 are public universities [27]. In addition, the Kenya human resources for health norms and standards guidelines for the health sector require that nurse specialists be deployed in all levels of health care except dispensaries and community units [10]. Establishing more MScN programs could be one of the ways to ensure adequate supply of MScN graduates for deployment in the various levels of health care. Establishing more MScN programs will increase access to higher nursing education therefore increasing the pool of highly qualified nurses that are a critical component of the health workforce [3]. This may help bridge the current gap in nursing workforce in Kenya. Specifically, there were 159.3 nurses per 100,000 population in Kenya in 2013 against a World health Organization recommendation of 228 per 100,000 population [6]. Our finding that a bigger majority of volunteers intended to pursue MScN and felt more MScN programs need to be established implies that the ongoing rollout of universal health coverage in Kenya needs to be accompanied by the expansion of higher education for nurses in various parts of the country. This will increase access to higher nursing education hence producing the highly qualified nursing workforce that is needed for the successful implementation of universal health coverage [3,28].Of interest, compared to volunteers who first studied nursing at diploma level then upgraded to degree, a bigger proportion of volunteers who directly enrolled for a degree in nursing after high school expressed a desire to pursue an MScN. Most of the nurses who upgraded their qualifications are already working. In contrast, nurses who directly enroll for the degree course after high school are not readily employed by the government. With the government being the major employer of the health work force in Kenya, it is possible that the frustrations of this category of nurses is pushing them to pursue further studies in order to qualify for more employment opportunities beyond the government.

The preferred nursing speciality was critical care/renal nursing. The government of Kenya rolled out the managed equipment services project in 2015. The project aimed at expanding access to specialized healthcare services by providing specialized hospital equipment including theatre and intensive care equipment [29]. The program led to an increase in critical care units which potentially led to an increase in demand for critical care nurses. Additionally, the COVID-19 pandemic led to efforts by the government of Kenya to expand critical care services with increased investment in equipment but there was still a shortfall in human resources [30]. It is possible that the increased interest in critical care among volunteers was driven by the increase in critical care units hence presenting potential job opportunities. Indeed, our findings suggest that a majority of the volunteers (n = 184, 54.6%) indicated that their motive for wanting to pursue MScN was to broaden career opportunities. In addition, the preferred learning mode was part- time. Majority of post graduate students in Kenya are working thus finance their own studies [31]. It is plausible that the part- time mode of study is preferred because it offers a flexible academic trajectory that allows students to pursue studies while working at the same time [32]. Most of the existing MScN programs are offered on part- time basis. Our finding that the part- time learning mode was the most preferred implies that Kenyan universities need to continue offering the MScN program on part- time basis. Further, Kenyan universities wishing to start the MScN program need to explore a variety of learning modes including part- time.

Almost 9 out of every 10 volunteers felt their skills were inferior to their job responsibilities. A previous study conducted in Zambia reported similar findings that 93% of workers in public health disciplines were not formerly trained for their roles [33] thus possessed skills that were inferior to their job responsibilities. The government of Kenya employs about 81.0% of the nursing workforce making it the main employer of nurses in Kenya [7]. However, only a few nurses with a master’s degree are employed by the national and county governments. Indeed, our findings show that only 55.1% of our volunteers reported having at least one colleague with an MScN qualification. The feeling amongst volunteers that they possessed skills that were inferior to their job responsibilities may suggest that nurses with a bachelor’s degree in Kenya are undertaking responsibilities meant for nurses with MScN qualifications. Indeed, our findings show that 48.3% of volunteers who were working indicated that there were junior nurses in their workplaces carrying out responsibilities that ought to be carried out by nurses with MScN qualification. Further, our findings show that all study volunteers had carried out at least one task at their workplace that is to be carried out by MScN graduates as per the scope of practice for nurses in Kenya [34]. A previous study from Kenya equally showed that nurses with lower education qualifications were undertaking advanced nursing practice roles [18]. Our findings imply that there is a need for government agencies both at national and county levels to collaborate and implement the scope of practice for nurses. This may require scaling up employment of nursing cadres that are in limited supply. Further, the finding that majority of participants felt that they possessed skills that were inferior to their job responsibilities may imply that future studies need to explore the quality of training for graduate nurses in Kenya.

We found no correlation between desire to pursue MScN and job satisfaction. A previous study that enrolled graduate nurses from Bangladesh equally reported the absence of a significant correlation between the desire to pursue MScN and job satisfaction [21]. However, our finding conflicts with previous studies that found that lower job satisfaction predicted a desire for higher nursing education [35,36]. Our finding might be due to the higher job satisfaction scores. All the same, an MScN program would increase the capacity and quality of patient care by increasing self-esteem, enhancing communication, promoting professional growth and enhancing the application of knowledge into practice all of which may potentially influence job satisfaction [37].

Our study is not without limitations. Data collection was done through an online survey. Although the link to the online form was shared through hospital managers and other contacts, it is possible that some nurses may have missed it hence had no chance of participating. This may have led to a convenience sample which then limits the generalizability of our findings. However, during data collection, investigators, who also doubled as faculty in their respective universities reached out to their former students who were interns, shared the link with them and encouraged them to share it with other nurses in their work places. Given that interns are usually posted to various hospitals all over the country, the act of reaching out to them is likely to have reduced the selection bias. Secondly, our study enrolled both nurse interns and nurses who are fully registered post-internship. Nurse interns are younger and fresh from university so are more likely to have a desire to pursue higher nursing education compared to their counterparts most of whom are older and with dependants hence may not be enthusiastic about further studies. Thirdly, all survey questions were made mandatory. This may have limited the liberty that participants usually have of not responding to questions they are not comfortable with and exiting the study at any point as they wish as provided for in ethical principles. This move was taken to minimize non- response that is relatively high in online surveys. Finally, some of the investigators work in universities that currently do not offer the MScN program and intend to offer the same in future therefore presenting a risk of bias based on their institutional need to start the program. However, 2 investigators actually are affiliated with universities that offer the program. Further, the authors had no control over how participants responded to the survey questions.

## Conclusion

Our findings suggest a strong desire by graduate nurses in Kenya to pursue MScN with a preference for critical care specialization while part-time is the preferred learning mode. Additionally, most volunteers felt their skills were inferior to their professional responsibilities. There was no correlation between intention to pursue MScN and job satisfaction. There is a need to establish more MScN programs in Kenya coupled with the deployment of nurses as per the scope of practice.

## Supporting information

S1 File Questionnaire. (DOCX)
