# Supplementary material for: Needs assessment for master of nursing program among Kenyan nurses
Source: PLoS One. 2025 May 5;20(5):e0322813. doi: 10.1371/journal.pone.0322813 (PMC12052190; doi:10.1371/journal.pone.0322813)
Supplement: S1 File — (DOCX) [file pone.0322813.s001.docx]

**APPENDIX 1: QUESTIONNAIRE**

1. Are you a Nurse registered in Kenya or a Nurse intern?
2. Are you a holder of a bachelor’s degree in Nursing (BScN), BSc Mid wifery or a BSc in any other nursing field?
3. Do you have or are you currently taking a master degree in Nursing?

If your answer to (a) and (b) above is “YES” but “No” to (c) then you are eligible. Please proceed to the survey questions below.

Instructions

Please try to answer all questions and to the best of your knowledge. Where you are not sure please take your best guess.

Section A: Sociodemographic details

1. What is your Date of birth [dd/mm/yyyy] …………………………………
2. Which is your home county…………...
3. In which county do you currently reside ………………….
4. Which sex do you identify yourself with (Male [ ] Female [ ] Intersex [ ])
5. What is your religion (Catholic [ ] Protestant [ ] Muslim [ ] Other [ ])
6. What is your marital status (Married [ ] Single [ ] Separated [ ] Divorced [ ] Widow/Widower [ ])
7. What level/s of nursing training have you undergone? Tick all that apply

Certificate [ ] Diploma [ ] Higher diploma [ ] Bachelor’s Degree [ ]

1. Are you currently enrolled for a training program in nursing? (Yes [ ] No [ ])
2. If Yes, what is the level of the training program

Certificate [ ] Diploma [ ] Postgraduate diploma [ ] Higher diploma [ ] Other [ ]

1. Are you a nurse intern? (Yes [ ] No [ ])
2. If not, what activity are you mainly involved in currently? (Full-time employment [ ] Part time employment/locum [ ] In business/self employed [ ] Looking for work [ ])
3. If employed, what is the name of the organization/health facility that you work for? ……………………………………
4. If employed, what is the type of the organization/health facility you are working for (Public [ ] Private [ ] faith based [ ])
5. If employed, in which county do you work in …………
6. What is your Designation (Nursing Officer [ ] Nurse manager [ ] Other [ ])
7. What is your working experience in years? …….
8. What are your current employment terms (Contract [ ] Permanent & pensionable [ ] Other [ ]
9. In which department do you work in (medical-surgical [ ] Maternity/Labour ward [ ] Pediatrics [ ]Theatres [ ] OPD/Emergency [ ] Others specify ………………………………..

Section B: Need for MScN

1. Would you pursue an MScN (Yes [ ] No [ ])
2. If so when (Immediately [ ] One year from now [ ] Two years from now [ ] Three years from now [ ] More than 3 years from now [ ] Don’t know [ ])
3. If so in which university would you like to pursue your MScN……………….
4. If so what is your motivation for studying MScN (Gaining professional knowledge/skills [ ] Broadening career opportunities [ ] Increasing chances of promotion [ ] N/A [ ] Other…………………………………….)
5. If so what are your preferred Nursing specialty areas for MScN (Nursing management [ ] Nursing education [ ] Community/Public Health Nursing [ ] Psychiatric Nursing [ ] Obstetric Nursing [ ] Pediatric Nursing [ ] Critical care [ ] Medical-Surgical Nursing [ ] Other specify………………………………………..)
6. If so what is your preferred teaching/learning style in the MScN program (Full time [ ] Part time [ ] Purely online [ ] Blended i.e. online and face to face [ ])
7. If so what are your career goals after MScN (Teaching [ ] Clinical [ ] Management [ ] Research [ ] Pursue PhD [ ] Other specify………………………………………………)
8. I so which emerging issues or new topic would you want to be included in the MScN program………………………….
9. Do you think there is need to start an MScN program at PU? (Yes [ ] No [ ])
10. Is there anyone with MScN training in your organization (Yes [ ] No [ ] Don’t know [ ])
11. If yes how many ……… (if not sure please take your best guess)
12. If yes, what is/are their area/s of specialization (Nursing management [ ] Nursing education [ ] Community/Public Health Nursing [ ] Psychiatric Nursing [ ] Obstetric Nursing [ ] Pediatric Nursing [ ] Critical care [ ] Medical-Surgical Nursing [ ] Other specify………………………………………..)
13. If no, would the organization you work for benefit from employing MScN graduates? (Yes [ ] No [ ])
14. If yes, which MScN specialty area would be needed most in your organization (Nursing management [ ] Nursing education [ ] Community/Public Health Nursing [ ] Psychiatric Nursing [ ] Obstetric Nursing [ ] Pediatric Nursing [ ] Critical care [ ] Medical-Surgical Nursing [ ] Other specify………………………………………..)
15. If MScN graduates would be employed, what would be their role in your organization (Offer clinical expertise [ ] Management [ ] Research [ ] Policy [ ] Mentorship to staff and students [ ] Other………………………………………..)

Section C: Skills mismatch

1. Do you feel that you have the skills to cope with more demanding duties than those you are required to perform in your current job? (Yes [ ] No [ ] )
2. Do you feel that you need further training in order to cope well with your present duties (Yes [ ] No [ ] )
3. Are there certificate or diploma nurses in your organization performing roles that ought to be a preserve of MScN graduates (Yes [ ] No [ ] I don’t know [ ]
4. How would you rate the existing skills gaps that MScN training may resolve (Very high [ ] Medium [ ] Low [ ])
5. Have you ever taken part in any of the following activities in your daily work? [Tick all that apply]

|  |  | **Yes** | **No** |
| --- | --- | --- | --- |
| 1 | Taking up the role of a consultant nurse |  |  |
| 2 | Providing local, national and regional leadership in nursing |  |  |
| 3 | Develop, manage and evaluate health programs in nursing |  |  |
| 4 | Interpret various health policies and facilitate their implementation |  |  |
| 5 | Develop policies and guidelines appropriate in a specific nursing field |  |  |
| 6 | Design, implement and evaluate mentorship programs to nurses, midwives and other health professionals |  |  |
| 7 | Analyze ethical issues in a specialized area of nursing and develop appropriate guidelines to address them |  |  |
| 8 | Conduct training needs assessment of staff in a specialized nursing area and develop appropriate human resource development programs. |  |  |
| 9 | Contribute in policy development is a specialized area of nursing locally, nationally, regionally and globally. |  |  |
| 10 | Design and conduct research to generate knowledge to inform practice and policy. |  |  |
| 11 | Publish and participate in scholarly activities |  |  |
| 12 | Support the profession’s growth in a specialized area of nursing and participation in educational programs |  |  |
| 13 | Advocate for increased budgetary allocation in health programs and in a specialized area of nursing |  |  |
| 14 | Plan budget and manage health finances |  |  |
| 15 | Prescribe and dispense drugs in a specialized area of nursing |  |  |
| 16 | Network with developmental and bilateral partners in resource mobilization for health |  |  |
| 17 | Develop proposals for resource mobilization |  |  |
| 18 | Set standards in conjunction with KEBS for medical supplies |  |  |

Section D: Job satisfaction

1. In this section we would like to assess how satisfied you are with your current job

|  |  | Strongly disagree | Disagree | Neither agree nor disagree | Agree | Strongly agree |
| --- | --- | --- | --- | --- | --- | --- |
| 1 | This job does not live up to my expectations |  |  |  |  |  |
| 2 | Knowing what I do now, I would apply for this job again |  |  |  |  |  |
| 3 | I often feel like resigning |  |  |  |  |  |
| 4 | I know that I am doing a really worthwhile job |  |  |  |  |  |
| 5 | I am satisfied with the relationship I have with my nursing colleagues |  |  |  |  |  |
|  | I am satisfied with the relationship I have with my immediate supervisor |  |  |  |  |  |
| 6 | I worry that this job is undermining my health |  |  |  |  |  |
| 7 | On the whole, I am satisfied with my working relationships with other members of health care team |  |  |  |  |  |
